# Comparison of safety of lecanemab and aducanumab: a real-world disproportionality analysis using the FDA adverse event reporting system

**DOI:** 10.3389/fphar.2025.1593989

**Published:** 2025-05-30

**Authors:** Lingti Kong, Xiaohu Yang, Jian Xu

**Affiliations:** ^1^ Department of Pharmacy, The First Affiliated Hospital of Bengbu Medical University, Bengbu, China; ^2^ School of Pharmacy, Bengbu Medical University, Bengbu, China; ^3^ Institute of Emergency and Critical Care Medicine, The First Affifiliated Hospital of Bengbu Medical University, Bengbu, China

**Keywords:** lecanemab, aducanumab, safety, gender, FAERS

## Abstract

**Objective:**

Studies on anti-Aβ drugs for the treatment of Alzheimer’s disease (AD) have garnered significant attention; however, their safety still requires further research and monitoring. Although recent studies have analyzed the adverse drug events (ADEs) of lecanemab and aducanumab separately, there is a lack of comparison between these two drugs, and no exploration of gender differences. This study aims to compare the adverse reaction signals of lecanemab and aducanumab, also exploring the differences between genders.

**Research design and methods:**

We analyzed ADEs reported by patients using lecanemab and aducanumab, using the FDA adverse event reporting system (FAERS). The data was classified using the preferred terms (PTs) and systemic organ categories (SOCs). Four positive signal detection algorithms were used, namely, the Ratio-to-Ratio (ROR), proportional reporting ratio (PRR), multi item gamma poisson shrinker (MGPS), and bayesian belief propagation neural network (BCPNN). Additionally, the time-to-onset of ADEs was also compared between the two drugs and between male and female patients.

**Results:**

A total of 1,409 ADE reports in which an anti-Aβ antibody drug was primarily suspected were included in the study, comprising 892 cases (63.31%) of lecanemab and 517 cases (36.69%) of aducanumab. For both lecanemab and aducanumab, only the SOC ‘nervous system disorders’ met the criteria for positive signal for all four algorithms. The number of positive PT signals related to lecanemab and aducanumab was 40 and 33, respectively. Among them, “cerebral microbleeds,” “amyloid protein related imaging abnormalities (ARIA),” and “central nervous system superficial squamous cell hyperplasia” all exhibited strong signals, regardless of drug or sex of the patient. Additionally, there were some differences in PT signals between male and female patients, and some new PT signals that were not included in the drug labels were identified. The median time-to-onset of lecanemab was shorter than that of aducanumab (33 days vs. 146 days).

**Conclusion:**

Four signal calculation methods were used to assess potential adverse reaction signals of lecanemab and aducanumab. This study identified some new PT signals and some PT signals showed gender differences. The median time-to-onset of ADEs due to lecanemab is shorter than that due to aducanumab.

## 1 Introduction

Alzheimer’s disease (AD) is the most common neurodegenerative disease of the central nervous system (Jack et al., 2018; Kamatham et al., 2024). The prevailing hypotheses regarding the pathogenesis of AD include abnormal deposition of β-amyloid protein (amyloid-β, Aβ), tau protein phosphorylation, and cholinergic damage (Prajapati et al., 2024; Kamatham et al., 2024; Yang and Qiu, 2024). With the increasing prevalence of AD and the escalating public health crisis, there is an urgent need to develop suitable interventions for AD prevention, disease onset delay, delaying progression, and symptom improvement (Grabher, 2018). However, current AD treatments are limited, primarily focusing on symptomatic management. Cholinesterase inhibitors, such as donepezil, rivastigmine, and galantamine, and NMDA receptor antagonists, including memantine, are two classes of medications that were commonly used in the management of AD (Zuliani et al., 2024; Caratelli et al., 2020; Swerdlow et al., 2023). Acting on the brain through distinct mechanisms, they can temporarily improve or stabilize the patients’ cognitive symptoms but cannot halt the disease progression (Varadharajan et al., 2023).

As scientific research deepens, novel drugs targeting the root causes of AD, such as therapeutic strategies against Aβ, are gradually emerging, and offer new hope for patients (Li et al., 2018; Ma et al., 2024). Aducanumab, which functions by removing Aβ from the brain, received accelerated approval from the Food and Drug Administration (FDA) in 2021 as the first anti-Aβ drug (Rabinovici, 2021). However, its true therapeutic benefits, the transparency of trial design, and the consistency of data interpretation are unclear (Heidebrink and Paulson, 2024). Lecanemab reduces the deposition of Aβ in the brain and slows disease progression (Chowdhury and Chowdhury, 2023), it significantly improved patients’ cognitive function, becoming the world’s first drug to demonstrate a notable inhibitory effect on AD progression during clinical trials (Cohen et al., 2023). It was approved for marketing in July 2023, also marking it the first fully approved anti-Aβ drug (Mahase, 2023). In July 2024, another anti-Aβ drug, donanemab-azbt, received FDA approval for the treatment of early symptomatic AD (Dyer, 2024). The drugs that have already been approved for use and the numerous ongoing clinical studies have highlighted the potential of anti-Aβ drugs in the treatment of AD (Li et al., 2023; Liu et al., 2023; Xiong et al., 2024).

In spite of their promising potentials, anti-Aβ drugs are not without controversy (Kwon, 2024). Their differences in efficacy have been demonstrated in clinical trials, and treatment-related adverse events, such as amyloid-related imaging abnormalities (ARIA), have attracted widespread attention (Belder et al., 2024; Terao and Kodama, 2024). This indicates that although these drugs have made breakthroughs in the field of AD treatment, their safety and efficacy still need further research and monitoring (Kwon, 2024; Terao and Kodama, 2024; Zhang et al., 2024; Wu et al., 2023).

Pharmacovigilance research is a crucial aspect of ensuring drug safety, providing clinical guidance for physicians and informing policymaking by drug regulatory authorities (Rong et al., 2024; Ali et al., 2024; Li et al., 2024; Deng et al., 2024). Currently, there are multiple databases for adverse drug reactions (ADRs) worldwide, such as the World Health Organization Adverse Drug Reaction Case Report Database (VigiBase), the US Food and Drug Administration Adverse Drug Reaction Database (FAERS), the European Adverse Drug Reaction Database (EudraVigilance), the United Kingdom National Adverse Drug Reaction Database (Yellow Card Scheme), the Canada Vigilance Adverse Reaction database (CVAR), etc. Among them, the FAERS is the most widely used database due to its large data volume and easy accessibility (Rong et al., 2024; Ali et al., 2024; Li et al., 2024; Jiang et al., 2024).

Given the complexity of AD treatment and the challenges of new drug development, pharmacovigilance research for anti-Aβ drugs is particularly important due to their novel mechanisms of action and relatively short period of market application (Sato et al., 2024). Although recent studies have analyzed the ADEs of lecanemab and aducanumab separately (Xing et al., 2025; Li et al., 2025; Wu et al., 2025), there is a lack of comparison between these two drugs, and no exploration of gender differences. The current study aims to compare the adverse reaction signals of lecanemab and aducanumab, while also exploring the differences between genders, which will contribute to enhancing the understanding of the safety profile and current knowledge of these two drugs.

## 2 Materials and methods

### 2.1 Data sources

**FIGURE 1:**
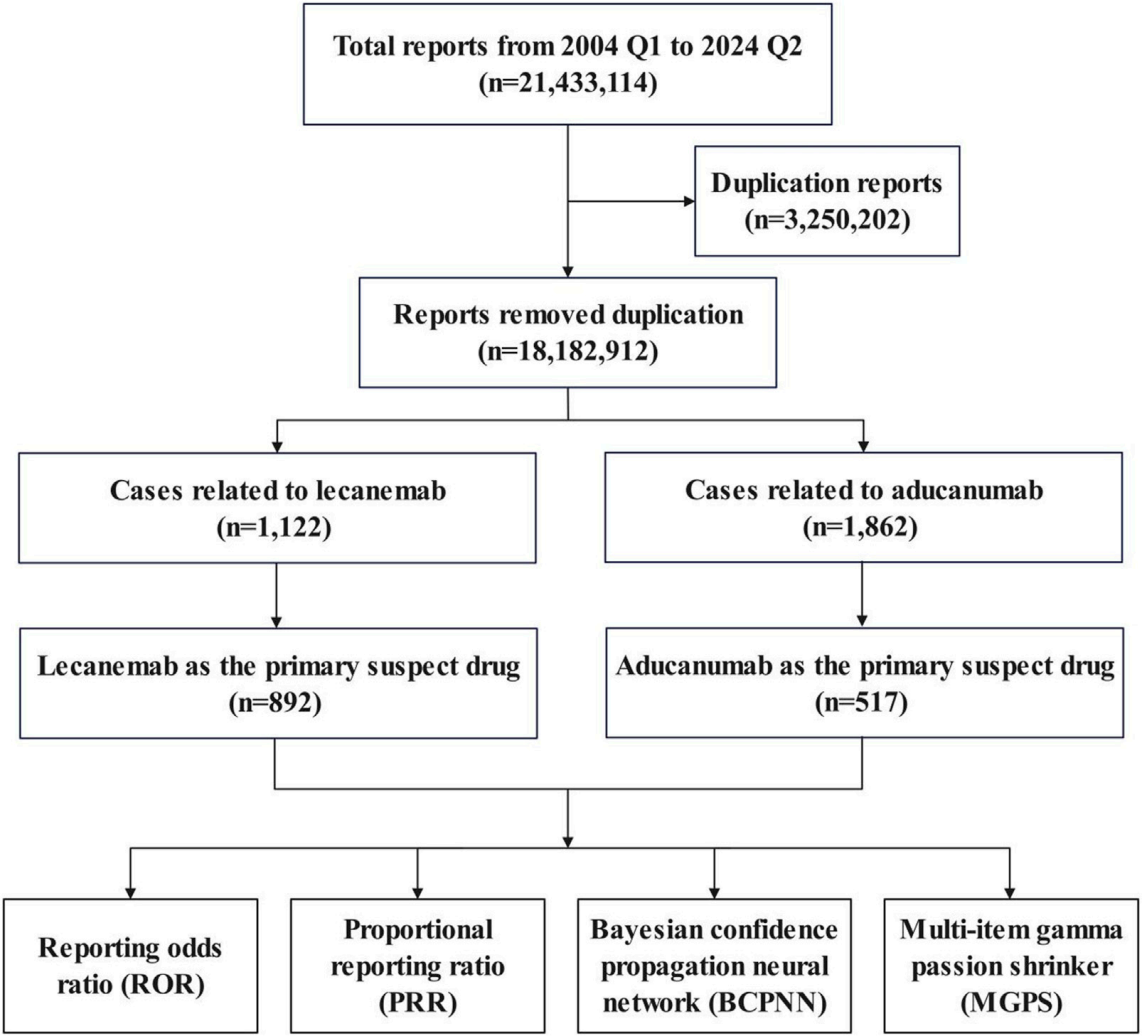
Data filtering flowchart.

The data for this study were sourced from the FAERS database (updated quarterly), selecting data from Q1st 2004 to Q2nd 2024. The dataset consisted of 7 data tables (DEMO, DRUG, REAC, OUTC, RPSR, THER, and INDI). The structure and content of these tables follow the International Council for Harmonisation (ICH) guidelines for safety reporting, and adverse reactions were coded using the Medical Dictionary for Regulatory Activities (MedDRA). Additionally, we removed duplicate data based on the case ID and primary ID. The data processing flow is shown in Figure 1.

### 2.2 Data filtering

While searching the database, both common names (“lecanemab-irmb,” “lecanemab,” “aducanumab-avwa,” “aducanumab”) and product names (“leqembi,” “aduhelm”) were used as keywords. Only ADEs whose role code was PS (primarily suspected) were included in this study. ADEs were described and classified using the preferred terms (PTs) and systemic organ categories (SOCs) from the terminology set in the Medical Dictionary of Adverse Drug Reactions (MedDRA v.26.0).

### 2.3 Data mining

Commonly used methods for the detection of adverse drug reaction signals include two categories and four specific algorithms (Fusaroli et al., 2024). One category is the frequency counting method, which includes Reporting Odds Ratio (ROR) and Proportional Reporting Ratio (PRR). The other category is the Bayesian method, which includes Multi-item Gamma Poisson Shrinker (MGPS), Bayesian Confidence Propagation Neural Network (BCPNN). These methods have their respective advantages and disadvantages, yet none of them stands out as superior. The frequency counting method (ROR, PRR) exhibit high sensitivity but low specificity, indicating a significant likelihood of false positives. On the other hand, the Bayesian method (MGPS, BCPNN) demonstrates greater robustness in detecting rare safety signals. Previous studies have suggested the use of at least one frequency counting method and one Bayesian method to minimize false positive signals (Fusaroli et al., 2024; Noguchi et al., 2021). In this study, only signals that simultaneously satisfy the threshold criteria of the four algorithms were regarded as positive signals. The calculation methods and threshold criteria of the four algorithms are shown in Table 1 (Jiang et al., 2024).

**TABLE 1:** Summary of the main algorithms used for signal detection.

Algorithm	Equation	Criteria
ROR	$ROR=\frac{a}{c}\times \frac{b}{d}$ $95\%CI=e^{\ln\left(ROR\right)\pm 1.96\sqrt{\frac{1}{a}+\frac{1}{b}+\frac{1}{c}+\frac{1}{d}}}$	lower limit of 95%CI > 1 a≥3
PRR	$PRR=\frac{a}{\left(a+b\right)}÷\frac{c}{\left(c+d\right)}$ $\chi^{2}=\frac{{\left(ad-bc\right)}^{2}\times \left(a+b+c+d\right)}{\left(a+b\right)\left(c+d\right)\left(a+c\right)\left(d+b\right)}$ $95\%CI=e^{\ln\left(PRR\right)\pm 1.96\sqrt{\frac{1}{a}-\frac{1}{a+b}+\frac{1}{c}-\frac{1}{c+d}}}$	PRR≥2 $\chi^{2}\geq 4,a\geq 3$
BCPNN	$V\left(IC\right)=\frac{1}{{\left(\ln\right)}^{2}}\left[\frac{b+c+d+\gamma -1}{\left(a+1\right)\backslash \left(1+a+b+c+d+\gamma \right)}+\frac{c+d+1}{\left(a+b+1\right)\left(a+b+c+d+3\right)}+\frac{b+d+1}{\left(a+c+1\right)\left(a+b+c+d+3\right)}\right]$ $\gamma =\frac{{\left(a+b+c+d+2\right)}^{2}}{\left(a+b+1\right)\left(a+c+1\right)}$ $E\left(IC\right)=\log_{2}\frac{\left(a+1\right){\left(a+b+c+d+2\right)}^{2}}{\left(a+b+c+d+\gamma \right)\left(a+b+1\right)\left(a+c+1\right)}$ $IC025=E\left(IC\right)-2\sqrt{V\left(IC\right)}$ $95\%CI=e^{\ln\left(IC\right)\pm 1.96\sqrt{\frac{1}{a}+\frac{1}{b}+\frac{1}{c}+\frac{1}{d}}}$	IC025>0
MGPS	$EBGM=\frac{a\left(a+b+c+d\right)}{\left(a+c\right)\left(a+b\right)}$ $EBGM05=e^{\ln\left(EBGM\right)-1.96\sqrt{\frac{1}{a}+\frac{1}{b}+\frac{1}{c}+\frac{1}{d}}}$ $95\%CI=e^{\ln\left(EBGM\right)\pm 1.96\sqrt{\frac{1}{a}+\frac{1}{b}+\frac{1}{c}+\frac{1}{d}}}$	EBGM05≥2 N>0

Abbreviations: ROR, reporting odds ratio; PRR, proportional reporting ratio; BCPNN, bayesian confidence propagation neural network; MGPS, multi-item gamma passion shrinker; IC, information component; EBGM, empirical Bayes geometric mean; a, number of reports arising from the suspect adverse events (AE) and the suspect drug; b, number of reports arising from the suspect AE and all other drugs; c, number of reports arising from the suspect drug and other ADEs; d, number of reports arising from other drugs and other ADEs; CI, confidence interval; χ^2^, chi-squared; IC025, lower limit of 95% two-sided CI of the IC; EBGM05, lower limit of 95% one-sided CI of EBGM.

Regarding the different types of drugs and sex-based variations in ADEs, we compared the time-to-onset of the ADE to facilitate differentiated medication monitoring. The calculation method for the time-to-onset is the interval between the onset date (EVENT_DT) and start date (START_DT), and excludes reports with missing or unreasonable dates.

## 3 Results

### 3.1 Descriptive characteristics

As shown in Table 2, a total of 1,409 ADEs related to anti-Aβ drugs were reported, of which 892 cases were related to lecanemab (63.31%) and 517 cases were related to aducanumab (36.69%). The number of reports from female and male patients were 747 cases (53.02%) and 568 cases (40.31%), respectively; additionally, 94 cases had missing gender information. The largest group of reporters were consumers (667 cases, 47.34%), followed by physicians (442 cases, 31.37%). Reporting countries were mainly from the United States (1,309 cases, 92.90%).

**TABLE 2:** Patients’ clinical characteristics.

Characteristics	Lecanemab	Aducanumab	Overall
	(N = 892)	(N = 517)	(N = 1,409)
Sex			
Female	488 (54.71%)	259 (50.10%)	747 (53.02%)
Male	338 (37.89%)	230 (44.49%)	568 (40.31%)
Missing	66 (7.40%)	28 (5.42%)	94 (6.67%)
Age (years)			
<18	3 (0.34%)	0 (0.00%)	3 (0.21%)
18–64.9	92 (10.31%)	29 (5.61%)	121 (8.59%)
65–85	602 (67.49%)	276 (53.38%)	878 (62.31%)
>85	23 (2.58%)	13 (2.51%)	36 (2.56%)
Missing	172 (19.28%)	199 (38.49%)	371 (26.33%)
Reporter type			
Consumer	455 (51.01%)	212 (41.01%)	667 (47.34%)
Health-professional	148 (16.59%)	114 (22.05%)	262 (18.59%)
Physician	259 (29.04%)	183 (35.40%)	442 (31.37%)
Pharmacist	18 (2.02%)	5 (0.97%)	23 (1.63%)
Missing	12 (1.35%)	3 (0.58%)	15 (1.06%)
Reporter country			
United States	833 (93.39%)	476 (92.07%)	1,309 (92.90%)
Japan	31 (3.48%)	11 (2.13%)	42 (2.98%)
France	4 (0.45%)	4 (0.77%)	8 (0.57%)
Italy	3 (0.34%)	3 (0.58%)	6 (0.43%)
Canada	1 (0.11%)	5 (0.97%)	6 (0.43%)
Switzerland	1 (0.11%)	5 (0.97%)	6 (0.43%)
China	5 (0.56%)	0 (0.00%)	5 (0.35%)
Spain	2 (0.22%)	3 (0.58%)	5 (0.35%)
Great Britain	3 (0.34%)	1 (0.19%)	4 (0.28%)
Korea	3 (0.34%)	0 (0.00%)	3 (0.21%)
Finland	0 (0.00%)	3 (0.58%)	3 (0.21%)
Argentina	2 (0.22%)	0 (0.00%)	2 (0.14%)
Australia	2 (0.22%)	0 (0.00%)	2 (0.14%)
Israel	1 (0.11%)	1 (0.19%)	2 (0.14%)
Sweden	1 (0.11%)	1 (0.19%)	2 (0.14%)
Germany	0 (0.00%)	2 (0.39%)	2 (0.14%)
Poland	0 (0.00%)	1 (0.19%)	1 (0.07%)
United Arab Emirates	0 (0.00%)	1 (0.19%)	1 (0.07%)

### 3.2 SOCs involved in positive signals

At the SOCs level, the frequency and signal strength of the ADEs involved in lecanemab and aducanumab are shown in Tables 3, 4, respectively. Both lecanemab and aducanumab involve 22 types of SOC, with 20 of them being the same. Furthermore, only the “Nervous system disorders” SOC simultaneously satisfied all four algorithmic positive criteria for both lecanemab and aducanumab.

**TABLE 3:** Frequency and signal strength of ADEs at the level of System Organ Classification (SOC) for lecanemab.

SOCs	Frequency	ROR (95% CI)	PRR (χ^2^)	EBGM (EBGM05)	IC (IC025)
Nervous system disorders	751	6.08 (5.56–6.64)	4.24 (2035.05)	4.24 (3.94)	2.09 (0.42)
General disorders and administration site conditions	475	1.40 (1.26–1.55)	1.31 (41.93)	1.31 (1.20)	0.39 (−1.28)
Psychiatric disorders	160	1.37 (1.17–1.61)	1.34 (14.95)	1.34 (1.17)	0.43 (−1.24)
Ear and labyrinth disorders	8	0.88 (0.44–1.77)	0.88 (0.12)	0.88 (0.49)	−0.18 (−1.85)
Gastrointestinal disorders	155	0.86 (0.73–1.01)	0.87 (3.49)	0.87 (0.76)	−0.21 (−1.87)
Vascular disorders	32	0.70 (0.50–1.00)	0.71 (3.90)	0.71 (0.53)	−0.50 (−2.16)
Eye disorders	28	0.67 (0.46–0.97)	0.67 (4.52)	0.67 (0.49)	−0.57 (−2.24)
Metabolism and nutrition disorders	26	0.58 (0.39–0.85)	0.58 (8.06)	0.58 (0.42)	−0.78 (−2.45)
Injury, poisoning and procedural complications	113	0.56 (0.46–0.67)	0.58 (37.6)	0.58 (0.50)	−0.78 (−2.45)
Musculoskeletal and connective tissue disorders	59	0.52 (0.40–0.67)	0.53 (25.91)	0.53 (0.43)	−0.91 (−2.58)
Investigations	67	0.50 (0.39–0.63)	0.51 (32.91)	0.51 (0.42)	−0.96 (−2.63)
Renal and urinary disorders	19	0.49 (0.31–0.77)	0.49 (10.00)	0.49 (0.34)	−1.02 (−2.68)
Cardiac disorders	27	0.48 (0.33–0.71)	0.49 (14.71)	0.49 (0.36)	−1.03 (−2.70)
Respiratory, thoracic and mediastinal disorders	45	0.44 (0.33–0.59)	0.45 (31.74)	0.45 (0.35)	−1.15 (−2.82)
Infections and infestations	48	0.42 (0.31–0.56)	0.43 (37.98)	0.43 (0.34)	−1.21 (−2.88)
Skin and subcutaneous tissue disorders	45	0.39 (0.29–0.52)	0.40 (42.20)	0.40 (0.31)	−1.31 (−2.98)
Social circumstances	2	0.22 (0.05–0.88)	0.22 (5.54)	0.22 (0.07)	−2.18 (−3.85)
Surgical and medical procedures	5	0.18 (0.07–0.42)	0.18 (19.15)	0.18 (0.09)	−2.49 (−4.15)
Hepatobiliary disorders	3	0.16 (0.05–0.49)	0.16 (13.57)	0.16 (0.06)	−2.66 (−4.33)
Neoplasms benign, malignant and unspecified (incl cysts and polyps)	7	0.12 (0.06–0.26)	0.13 (43.19)	0.13 (0.07)	−2.98 (−4.65)
Reproductive system and breast disorders	2	0.12 (0.03–0.46)	0.12 (13.46)	0.12 (0.04)	−3.10 (−4.76)
Blood and lymphatic system disorders	3	0.08 (0.03–0.26)	0.08 (30.14)	0.08 (0.03)	−3.56 (−5.23)

**TABLE 4:** Frequency and signal strength of ADEs at the level of System Organ Classification (SOC) for aducanumab.

SOCs	Frequency	ROR (95% CI)	PRR (χ^2^)	EBGM (EBGM05)	IC (IC025)
Nervous system disorders	613	12.78 (11.37–14.37)	6.39 (3,042.57)	6.38 (5.79)	2.67 (1.01)
Ear and labyrinth disorders	8	1.63 (0.81–3.27)	1.63 (1.94)	1.63 (0.91)	0.70 (−0.97)
Psychiatric disorders	81	1.27 (1.02–1.6)	1.25 (4.43)	1.25 (1.04)	0.33 (−1.34)
Injury, poisoning and procedural complications	89	0.83 (0.67–1.03)	0.84 (2.84)	0.84 (0.70)	−0.24 (−1.91)
Cardiac disorders	23	0.76 (0.51–1.16)	0.77 (1.63)	0.77 (0.54)	−0.38 (−2.05)
Neoplasms benign, malignant and unspecified (incl cysts and polyps)	21	0.70 (0.45–1.07)	0.7 (2.73)	0.7 (0.49)	−0.51 (−2.18)
Infections and infestations	41	0.67 (0.49–0.91)	0.68 (6.56)	0.68 (0.52)	−0.56 (−2.23)
Metabolism and nutrition disorders	16	0.65 (0.4–1.07)	0.66 (2.90)	0.66 (0.44)	−0.60 (−2.27)
Renal and urinary disorders	13	0.62 (0.36–1.07)	0.62 (3.01)	0.62 (0.39)	−0.68 (−2.35)
Vascular disorders	14	0.57 (0.33–0.96)	0.57 (4.59)	0.57 (0.37)	−0.81 (−2.47)
Hepatobiliary disorders	5	0.48 (0.20–1.16)	0.49 (2.75)	0.49 (0.23)	−1.04 (−2.71)
Gastrointestinal disorders	40	0.39 (0.28–0.54)	0.41 (36.7)	0.41 (0.32)	−1.28 (−2.95)
Eye disorders	8	0.35 (0.17–0.70)	0.35 (9.59)	0.35 (0.20)	−1.50 (−3.16)
General disorders and administration site conditions	76	0.34 (0.27–0.43)	0.39 (89.91)	0.39 (0.32)	−1.37 (−3.04)
Musculoskeletal and connective tissue disorders	21	0.34 (0.22–0.52)	0.35 (27.1)	0.35 (0.24)	−1.52 (−3.19)
Investigations	24	0.32 (0.22–0.49)	0.34 (32.97)	0.34 (0.24)	−1.56 (−3.23)
Skin and subcutaneous tissue disorders	19	0.30 (0.19–0.47)	0.31 (30.24)	0.31 (0.21)	−1.67 (−3.34)
Respiratory, thoracic and mediastinal disorders	9	0.16 (0.08–0.31)	0.17 (39.62)	0.17 (0.10)	−2.59 (−4.26)
Immune system disorders	2	0.16 (0.04–0.63)	0.16 (9.01)	0.16 (0.05)	−2.65 (−4.32)
Blood and lymphatic system disorders	3	0.15 (0.05–0.48)	0.16 (13.90)	0.16 (0.06)	−2.68 (−4.35)
Product issues	2	0.11 (0.03–0.45)	0.11 (14.05)	0.11 (0.04)	−3.14 (−4.81)
Reproductive system and breast disorders	1	0.11 (0.02–0.76)	0.11 (7.46)	0.11 (0.02)	−3.22 (−4.88)

### 3.3 Positive PT signals

At the PT level, signals that satisfied all four algorithms simultaneously were considered positive signals; the frequency and signal strength of positive signals related to lecanemab and aducanumab are shown in Tables 5, 6, respectively.

**TABLE 5:** Frequency and signal strength of positive preferred term (PT) signals for lecanemab.

PTs	Frequency	ROR (95% CI)	PRR (χ^2^)	EBGM (EBGM05)	IC (IC025)
Amyloid related imaging abnormalities	31	40,653.32 (23,133.34–71,442)	40,047.73 (486,830.92)	15,705.60 (9,798.82)	13.94 (12.22)
Amyloid related imaging abnormality-oedema/effusion	95	15,974.80 (12,349.98–20,663.54)	15,245.58 (910,748.70)	9,588.41 (7,730.82)	13.23 (11.55)
Amyloid related imaging abnormality-microhaemorrhages and haemosiderin deposits	72	15,539.90 (11,585.22–20,844.53)	15,002.27 (683,280.18)	9,491.60 (7,423.68)	13.21 (11.53)
Superficial siderosis of central nervous system	5	2,877.72 (1,141.16–7,256.89)	2,870.81 (12,909.64)	2,583.82 (1,191.64)	11.34 (9.62)
Cerebral microhaemorrhage	4	452.17 (168.11–1,216.24)	451.31 (1766.39)	443.58 (193.82)	8.79 (7.12)
Brain fog	9	37.62 (19.54–72.44)	37.46 (318.99)	37.41 (21.62)	5.23 (3.56)
Slow speech	3	37.43 (12.05–116.23)	37.37 (106.05)	37.32 (14.46)	5.22 (3.55)
Infusion related reaction	64	29.89 (23.30–38.34)	29.00 (1730.24)	28.97 (23.52)	4.86 (3.19)
Chills	107	27.35 (22.52–33.23)	26.00 (2,574.5)	25.97 (22.07)	4.70 (3.03)
Brain oedema	11	25.49 (14.09–46.11)	25.36 (257.17)	25.33 (15.43)	4.66 (3.00)
Feeling cold^a^	19	19.60 (12.47–30.79)	19.43 (331.97)	19.41 (13.3)	4.28 (2.61)
Screaming^a^	4	18.21 (6.83–48.59)	18.18 (64.90)	18.17 (7.99)	4.18 (2.52)
Encephalitis^a^	3	13.00 (4.19–40.34)	12.98 (33.16)	12.97 (5.03)	3.70 (2.03)
Confusional state	70	12.82 (10.10–16.27)	12.43 (737.06)	12.42 (10.17)	3.63 (1.97)
Formication	3	12.05 (3.88–37.40)	12.03 (30.34)	12.03 (4.66)	3.59 (1.92)
Hiccups^a^	3	11.02 (3.55–34.20)	11.01 (27.28)	11.00 (4.26)	3.46 (1.79)
Incontinence	4	10.84 (4.06–28.91)	10.82 (35.64)	10.82 (4.76)	3.44 (1.77)
Headache	200	10.04 (8.68–11.62)	9.17 (1,471.39)	9.17 (8.12)	3.20 (1.53)
Cerebral haemorrhage	12	9.74 (5.52–17.19)	9.69 (93.58)	9.69 (6.03)	3.28 (1.61)
Influenza like illness	27	9.20 (6.29–13.44)	9.09 (194.61)	9.09 (6.61)	3.18 (1.52)
Sluggishness	3	8.44 (2.72–26.21)	8.43 (19.65)	8.43 (3.27)	3.08 (1.41)
Poor quality sleep^a^	6	8.22 (3.69–18.32)	8.20 (37.93)	8.20 (4.19)	3.04 (1.37)
Poor venous access	3	8.02 (2.59–24.91)	8.01 (18.42)	8.01 (3.11)	3.00 (1.33)
Status epilepticus	3	7.91 (2.55–24.56)	7.90 (18.09)	7.90 (3.06)	2.98 (1.31)
Head discomfort	4	6.73 (2.52–17.94)	6.71 (19.46)	6.71 (2.95)	2.75 (1.08)
Body temperature increased^a^	5	6.70 (2.78–16.11)	6.68 (24.17)	6.68 (3.21)	2.74 (1.07)
Anger^a^	8	6.67 (3.33–13.36)	6.65 (38.43)	6.65 (3.72)	2.73 (1.07)
Tremor^a^	38	6.63 (4.81–9.14)	6.52 (178.21)	6.52 (4.99)	2.71 (1.04)
Abnormal dreams^a^	6	6.07 (2.72–13.52)	6.05 (25.32)	6.05 (3.10)	2.60 (0.93)
Subdural haematoma	3	5.97 (1.92–18.52)	5.96 (12.39)	5.96 (2.31)	2.58 (0.91)
Pyrexia^a^	68	5.74 (4.51–7.31)	5.59 (257.69)	5.59 (4.57)	2.48 (0.82)
Disorientation	8	5.72 (2.85–11.45)	5.70 (31.00)	5.70 (3.19)	2.51 (0.84)
Aphasia	6	5.68 (2.55–12.65)	5.66 (23.05)	5.66 (2.90)	2.50 (0.83)
Generalised tonic-clonic seizure	3	5.66 (1.82–17.57)	5.66 (11.50)	5.65 (2.19)	2.50 (0.83)
Lacrimation increased^a^	5	5.13 (2.13–12.33)	5.12 (16.57)	5.12 (2.45)	2.36 (0.69)
Cognitive disorder	8	5.02 (2.51–10.06)	5.01 (25.67)	5.01 (2.80)	2.32 (0.66)
Lethargy	9	4.50 (2.34–8.65)	4.48 (24.36)	4.48 (2.59)	2.16 (0.50)
Somnolence^a^	29	4.22 (2.92–6.08)	4.17 (70.13)	4.17 (3.07)	2.06 (0.39)
Fatigue	97	3.74 (3.05–4.58)	3.61 (185.37)	3.61 (3.04)	1.85 (0.18)
Dizziness	57	3.38 (2.60–4.40)	3.32 (92.92)	3.31 (2.66)	1.73 (0.06)

Note:

^a^ Indicates that the PT is not included in the drug label.

**TABLE 6:** Frequency and signal strength of positive preferred term (PT) signals for aducanumab.

PTs	Frequency	ROR (95% CI)	PRR (χ^2^)	EBGM (EBGM05)	IC (IC025)
Amyloid related imaging abnormality-oedema/effusion	161	94,135.22 (72,490.24–122,243.21)	80,711.30 (4,822,088.11)	29,952.09 (24,070.25)	14.87 (13.19)
Amyloid related imaging abnormality-microhaemorrhages and haemosiderin deposits	114	73,646.19 (55,084.74–98,462.13)	66,209.92 (3,157,723.83)	27,700.65 (21,725.10)	14.76 (13.08)
Superficial siderosis of central nervous system	21	35,140.45 (19,978.72–61,808.33)	34,486.84 (420,025.69)	20,002.78 (12,470.67)	14.29 (12.57)
Amyloid related imaging abnormalities	14	18,246.40 (9,837.2–33,844.08)	18,020.15 (183,008.03)	13,073.72 (7,796.48)	13.67 (11.96)
Cerebral microhaemorrhage	7	1,484.30 (698.01–3,156.33)	1,475.10 (10,001.96)	1,430.81 (761.06)	10.48 (8.80)
Product name confusion	3	130.11 (41.83–404.66)	129.77 (382.29)	129.42 (50.08)	7.02 (5.35)
Brain oedema	14	60.24 (35.55–102.08)	59.50 (804.46)	59.43 (38.23)	5.89 (4.22)
Subarachnoid haemorrhage	8	41.57 (20.73–83.35)	41.28 (314.20)	41.24 (23.04)	5.37 (3.70)
Cerebral haemorrhage	27	41.18 (28.11–60.34)	40.22 (1,032.38)	40.19 (29.19)	5.33 (3.66)
Posterior reversible encephalopathy syndrome	3	18.12 (5.83–56.27)	18.07 (48.37)	18.07 (7.00)	4.18 (2.51)
Cognitive disorder	13	15.16 (8.78–26.20)	15.00 (169.93)	14.99 (9.49)	3.91 (2.24)
Head injury	7	11.92 (5.67–25.07)	11.86 (69.61)	11.85 (6.36)	3.57 (1.90)
Confusional state	34	11.44 (8.13–16.09)	11.12 (313.97)	11.12 (8.36)	3.47 (1.81)
Subdural haematoma	3	11.01 (3.55–34.21)	10.99 (27.24)	10.99 (4.26)	3.46 (1.79)
Haemorrhage intracranial	3	10.29 (3.31–31.96)	10.27 (25.09)	10.26 (3.98)	3.36 (1.69)
Skin cancer^a^	4	10.00 (3.75–26.69)	9.97 (32.27)	9.96 (4.38)	3.32 (1.65)
Seizure	20	9.93 (6.38–15.45)	9.77 (157.71)	9.77 (6.75)	3.29 (1.62)
Mental status changes	5	9.66 (4.01–23.27)	9.63 (38.66)	9.62 (4.61)	3.27 (1.60)
Ischaemic stroke^a^	3	8.94 (2.88–27.75)	8.91 (21.08)	8.91 (3.45)	3.16 (1.49)
Prescribed underdose	3	8.15 (2.63–25.32)	8.13 (18.78)	8.13 (3.15)	3.02 (1.36)
Post procedural complication	3	8.11 (2.61–25.17)	8.09 (18.64)	8.09 (3.13)	3.02 (1.35)
Transient ischaemic attack^a^	5	7.69 (3.19–18.51)	7.66 (28.96)	7.66 (3.67)	2.94 (1.27)
Aphasia	4	6.98 (2.61–18.63)	6.96 (20.42)	6.96 (3.06)	2.80 (1.13)
Cerebral infarction	3	6.46 (2.08–20.07)	6.45 (13.81)	6.45 (2.50)	2.69 (1.02)
Dementia	3	6.05 (1.95–18.79)	6.04 (12.61)	6.04 (2.34)	2.59 (0.93)
Breast cancer female^a^	3	5.65 (1.82–17.54)	5.63 (11.44)	5.63 (2.18)	2.49 (0.83)
Atrial fibrillation^a^	10	5.50 (2.95–10.25)	5.46 (36.46)	5.46 (3.24)	2.45 (0.78)
Urinary incontinence	3	5.34 (1.72–16.59)	5.33 (10.56)	5.33 (2.07)	2.41 (0.75)
Disorientation	4	5.27 (1.97–14.05)	5.25 (13.77)	5.25 (2.31)	2.39 (0.72)
Memory impairment	13	4.99 (2.89–8.63)	4.95 (41.02)	4.95 (3.13)	2.31 (0.64)
Vertigo	5	4.33 (1.80–10.43)	4.32 (12.76)	4.32 (2.07)	2.11 (0.44)
Fall	23	3.72 (2.46–5.62)	3.67 (44.82)	3.67 (2.59)	1.87 (0.21)
Headache	42	3.65 (2.68–4.97)	3.55 (77.74)	3.55 (2.74)	1.83 (0.16)

Note:

^a^ Indicates that the PT is not included in the drug label.

### 3.4 Positive PT signals differences between genders

**FIGURE 2:**
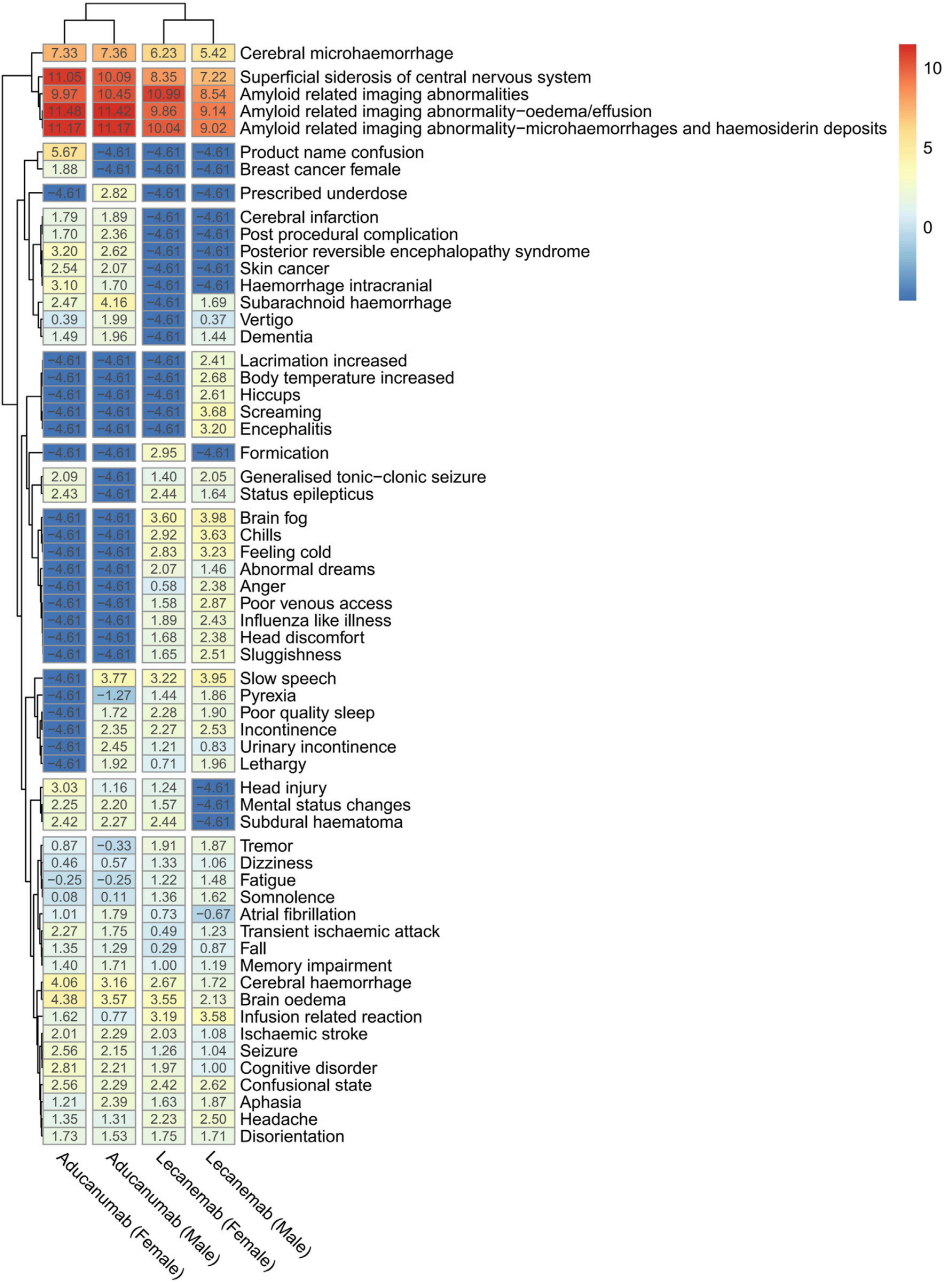
Cluster heatmap of positive PT signal differences between lecanemab and aducanumab at different genders.

There are differences in the prevalence of AD between female and male individuals; therefore, a log transformation of the ROR values was used to facilitate the identification and comparison of any sex-based differences in the signals of adverse effects between the same drug and between different drugs. A cluster heat map was then generated based on these values (with a value of 0.01 assigned in cases where the value was 0), as shown in Figure 2. “Cerebral microhemorrhage,” “Amyloid-related imaging abnormalities,” and “Superficial siderosis of the central nervous system” all exhibited strong signals.

### 3.5 Time-to-onset differences between genders

**FIGURE 3:**
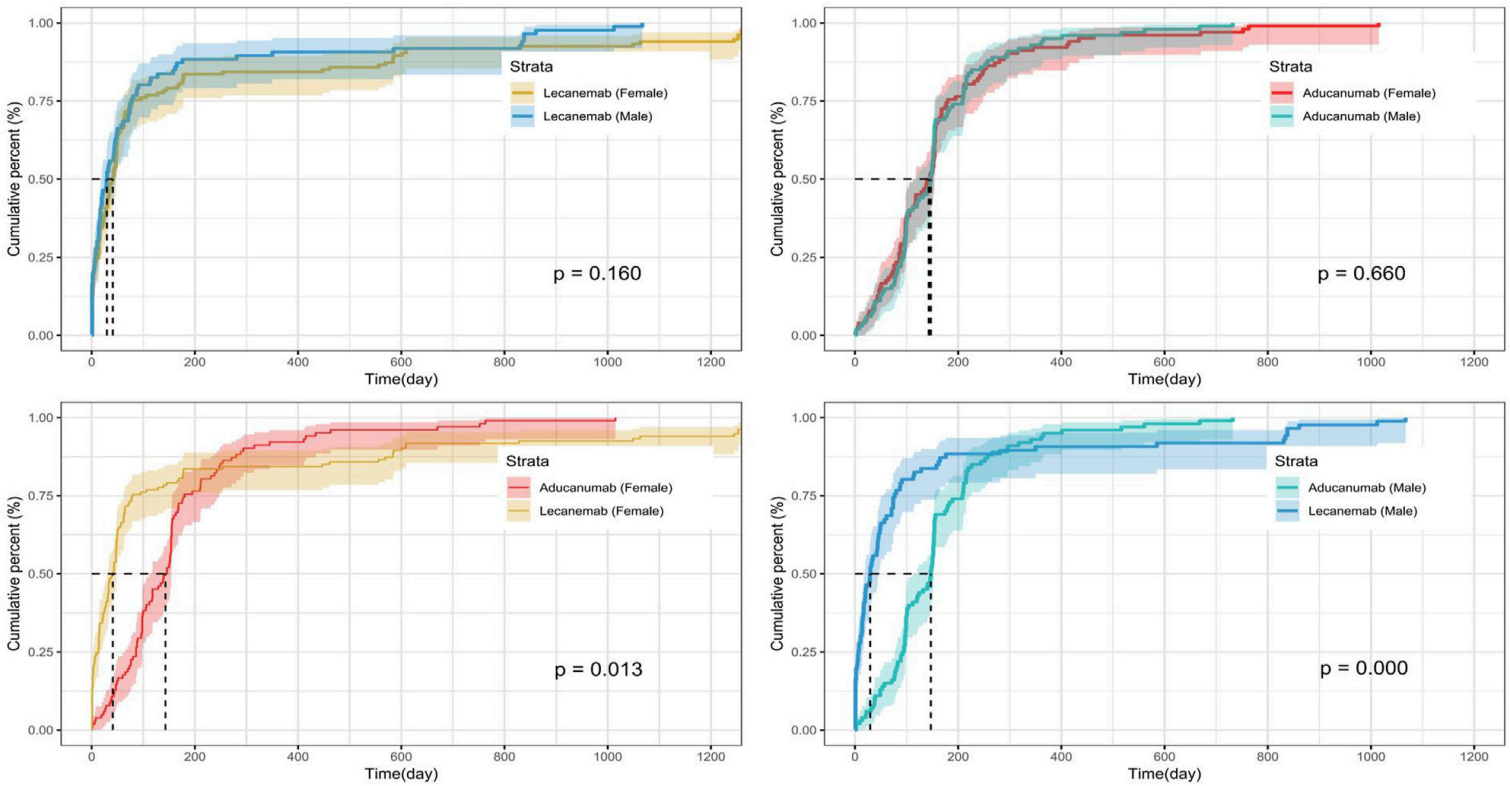
Time-to-onset of ADEs in lecanemab and aducanumab at different genders.

We calculated the time-to-onset of ADEs between the different drugs and between male and female patients (Figure 3). The median time-to-onset of ADEs among patients receiving lecanemab was shorter than that among those receiving aducanumab (33 days vs. 146 days). The median time-to-onset of ADEs varied across the treatment groups. For individuals receiving lecanemab, the median time-to-onset was 41 days for females and 29.5 days for males. Meanwhile, for those treated with aducanumab, the median time-to-onset was longer, at 143 days for females and 147 days for males.

## 4 Discussion

Anti-Aβ drugs are increasingly being used in the treatment of AD, In addition to the already marketed drugs, such as aducanumab, lecanemab, and donanemab, phase III clinical trials of drugs, such as remternetug, and AHEAD 3–45 are currently ongoing (Maheshwari et al., 2024; Rissman et al., 2024). Therefore, this study, which aims to compare the adverse reaction signals of lecanemab and aducanumab, will aid in improving the safety profile and current understanding of these two drugs, also serve as a reference for similar drugs during the phases of research and development (Sato et al., 2024).

As of the second quarter of 2024, a total of 1,409 cases of ADE primarily suspected to be due to anti-Aβ drugs were reported in the FAERS database. These included 892 reports for lecanemab (63.31%) and 517 reports for aducanumab (36.69%). For both lecanemab and aducanumab, the ADEs involved 22 types of SOC (Tables 3, 4). However, only one SOC (“Nervous system disorders”) simultaneously met all four algorithm positive criteria, and it had the highest number of ADEs among all types of SOC, suggesting a need for particular focus on the adverse reactions associated with this SOC.

The number of positive PT signals related to lecanemab and aducanumab was 40 and 33, respectively (Tables 5, 6). For lecanemab, “Headache,” “Chills,” and “Fatigue” were the three most common PTs in terms of frequency. In terms of signal intensity, four positive PTs had an ROR greater than 1,000, namely, “Amyloid-related imaging abnormalities,” “Amyloid-related imaging abnormality-edema/effusion,” “Amyloid-related imaging abnormality-microhemorrhages and hemosiderin deposits,” and “Cerebral microhemorrhage.” For aducanumab, “Amyloid-related imaging abnormality-edema/effusion,” “Amyloid-related imaging abnormality-microhemorrhages and hemosiderin deposits,” and “Headache” were the three most common PTs in terms of frequency. In terms of signal intensity, five positive PTs had an ROR greater than 1,000, namely, “Amyloid-related imaging abnormality-edema/effusion,” “Amyloid-related imaging abnormality-microhemorrhages and hemosiderin deposits,” “Superficial siderosis of the central nervous system,” “Amyloid-related imaging abnormalities,” and “Cerebral microhemorrhage.”

There are similarities and differences in the PT signals between two drugs. For example, “Cerebral infarction,” “Post procedural complication,” “Posterior reversible encephalopathy syndrome,” and “Skin cancer” only present signals in aducanumab, while “Brain fog,” “Chills,” “Feeling cold,” “Abnormal dreams,” “Anger,” and “Poor venous access” were observed only in lecanemab. However, ARIA (“Amyloid-related imaging abnormalities,” “Amyloid-related imaging abnormality-edema/effusion,” and “Amyloid-related imaging abnormality-microhemorrhages and hemosiderin deposits”) showed strong signals in both drugs (Sato et al., 2024). In addition, patients receiving lecanemab treatment had a shorter median time-to-onset of ADEs compared to those receiving aducanumab treatment (33 days vs. 146 days). The results of this study suggest that differentiated monitoring should be provided when using these two drugs in clinical practice.

This study also identified some positive PT signals that have not yet been included in drug labels. For lecanemab, new PT signals such as “Feeling cold,” “Screaming,” “Encephalitis,” “Hiccups,” “Poor quality sleep,” and “Increased lacrimation” were identified. For aducanumab, severe PT signals such as “Skin cancer,” “Breast cancer,” “Ischemic stroke,” and “Aerial fibrosis” were found. On one hand, some PTs may be related to the immune decline caused by anti-Aβ drugs. Studies have shown that Aβ deposition plays a positive role in the immune response of the body, and the removal of Aβ by lecanemab or aducanumab can lead to a decrease in immune function and an increased risk of infection (Abbott, A. 2020; Eimer et al., 2018). On the other hand, some PTs may be associated with the patient’s comorbidities. It is worth noting that, semagacestat, a γ-secretase inhibitor, also showed potential in the treatment of AD, but due to its increased risk of skin cancer in patients and poor efficacy, its phase III clinical trial was terminated prematurely (Karran and De Strooper, 2022). Overall, although the pharmacological mechanisms underlying these PTs are not fully understood, they deserve the attention of clinical and basic researchers to provide targeted pharmaceutical monitoring or optimize their structures in the future, which is one of the main objectives of this pharmacovigilance research (Thussu et al., 2024).

There are significant sex-based differences in AD, primarily reflected in a substantially higher number of female patients compared to male patients (Aggarwal and Mielke, 2023; Nebel et al., 2018). This may be related to various factors, such as estrogen levels, psychological factors, lifestyle, and social factors (Lopez-Lee et al., 2024). The difference in the number of ADE reports as shown in Table 2 is also consistent with this background, with 747 cases (53.02%) and 568 cases (40.31%) reported by female and male patients, respectively. It is unclear whether there are differences in adverse reactions between female and male patients when such drugs are used therapeutically. Therefore, a subgroup analysis was conducted, revealing sex-based differences in ADEs for both drugs (Figure 2). For patients receiving aducanumab, “Slow speech Pyrexia,” “Poor quality sleep,” “Incontinence,” “Urinary incontinence,” and “Lethargy” were reported by only male patients, while “Generalized tonic-clonic seizure” and “Status epilepticus” were reported by only female patients. For patients receiving lecanemab, “Increased lacrimation,” “Increased body temperature,” “Hiccups,” “Screaming,” and “Encephalitis” were reported only by male patients, while “Formication,” “Head injury,” “Mental status changes,” and “Subdural hematoma” were reported only by female patients. Moreover, there was no significant difference in the time-to-onset of ADEs between females and males receiving the same drug (Figure 3, P > 0.05). A deeper understanding of these differences and their causes is needed so that more targeted prevention and intervention measures can be developed in clinical practice to improve treatment compliance and the clinical effectiveness of AD (Lopez-Lee et al., 2024; Demetrius et al., 2021).

Some studies have shown that the actual benefits of Aβ monoclonal antibodies are minimal (Espay et al., 2024; Heidebrink and Paulson, 2024; de la Torre and Gonzalez-Lima, 2021); moreover, there are also concerning and poorly understood medication risks and the relatively high treatment costs (Alves et al., 2023; Espay et al., 2024; Nguyen et al., 2024; de la Torre and Gonzalez-Lima, 2021). If clinicians do not strictly screen patients according to the inclusion criteria of phase III clinical trials, the dangers of drug side effects may be amplified (Filippi et al., 2023). Therefore, for lecanemab and aducanumab, continuous monitoring and evaluation of their long-term safety are necessary. However, the successful launch of lecanemab and aducanumab marks the entry of anti-Aβ monoclonal antibodies into the mainstream drug queue for the treatment of AD, following acetylcholinesterase inhibitors and NMDA receptor antagonists, offering more treatment choices for patients with AD (Yang and Qiu, 2024; Varadharajan et al., 2023).

This study has several limitations. Firstly, the FAERS database is a spontaneous reporting system that can only indicate a correlation between drugs and adverse reactions, rather than a causal relationship, and it may be subject to shortcomings, such as incorrect reporting, non-standardized reports, influence from drug labeling, and confusion regarding the disease itself or its complications. Secondly, this study only utilized the FAERS database, which may introduce population bias when compared to data from other databases like VigiBase or EudraVigilance. Thirdly, due to the relatively short time since the introduction of these drugs, the reported number of adverse drug reactions is relatively small, which can also lead to some research deviations. In the future, it is necessary to encourage healthcare professionals and patients to actively and standardly report adverse reactions when using aducanumab, lecanemab, and other upcoming novel drugs, in order to expand the data scale to address these limitations.

## 5 Conclusion

In this study, four signal calculation methods (ROR, PRR, BCPNN, and MGPS) were used to assess the potential adverse reaction signals of lecanemab and aducanumab using the FAERS database. “Cerebral microbleeds,” “amyloid protein related imaging abnormalities (ARIA),” and “central nervous system superficial squamous cell hyperplasia” all exhibit strong signals between these two drugs. This study identified some new PT signals that are not currently listed in the drugs’ labels; furthermore, some PT signals showed sex-based differences. The median time-to-onset of ADEs for lecanemab was shorter than that for aducanumab. This study’s findings will promote the safe use of these two drugs.

## Data Availability

The original contributions presented in the study are included in the article/supplementary material, further inquiries can be directed to the corresponding authors.

## References

[B1] Abbott A. (2020). Are infections seeding some cases of Alzheimer's disease? Nature 587 (7832), 22–25. 10.1038/d41586-020-03084-9 33149296

[B2] Aggarwal N. T. Mielke M. M. (2023). Sex differences in Alzheimer's disease. Neurol. Clin. 41 (2), 343–358. 10.1016/j.ncl.2023.01.001 37030962 PMC10321561

[B3] Ali Z. Ismail M. Rehman I. U. Goh K. W. Razi P. Ming L. C. (2024). Association of anxiolytic drugs with Torsade de Pointes: a pharmacovigilance study of the Food and Drug Administration Adverse Event Reporting System. J. Pharm. Policy Pract. 17 (1), 2399716. 10.1080/20523211.2024.2399716 39291052 PMC11407426

[B4] Alves F. Kalinowski P. Ayton S. (2023). Accelerated brain volume loss caused by anti-beta-amyloid drugs: a systematic review and meta-analysis. Neurology 100 (20), e2114–e2124. 10.1212/WNL.0000000000207156 36973044 PMC10186239

[B5] Belder C. R. S. Boche D. Nicoll J. A. R. Jaunmuktane Z. Zetterberg H. Schott J. M. (2024). Brain volume change following anti-amyloid beta immunotherapy for Alzheimer's disease: amyloid-removal-related pseudo-atrophy. Lancet Neurol. 23 (10), 1025–1034. 10.1016/S1474-4422(24)00335-1 39304242

[B6] Caratelli V. Ciampaglia A. Guiducci J. Sancesario G. Moscone D. Arduini F. (2020). Precision medicine in Alzheimer's disease: an origami paper-based electrochemical device for cholinesterase inhibitors. Biosens. Bioelectron. 165, 112411. 10.1016/j.bios.2020.112411 32729530

[B7] Chowdhury S. Chowdhury N. S. (2023). Novel anti-amyloid-beta (Aβ) monoclonal antibody lecanemab for Alzheimer's disease: a systematic review. Int. J. Immunopathol. Pharmacol. 37, 3946320231209839. 10.1177/03946320231209839 37902139 PMC10617290

[B8] Cohen S. van Dyck C. H. Gee M. Doherty T. Kanekiyo M. Dhadda S. (2023). Lecanemab clarity AD: quality-of-life results from a randomized, double-blind phase 3 trial in early Alzheimer's disease. J. Prev. Alzheimers Dis. 10 (4), 771–777. 10.14283/jpad.2023.123 37874099

[B9] de la Torre J. C. Gonzalez-Lima F. (2021). The FDA approves aducanumab for Alzheimer's disease, raising important scientific Questions1. J. Alzheimers Dis. 82 (3), 881–882. 10.3233/JAD-210736 34250943

[B10] Demetrius L. A. Eckert A. Grimm A. (2021). Sex differences in Alzheimer's disease: metabolic reprogramming and therapeutic intervention. Trends Endocrinol. Metab. 32 (12), 963–979. 10.1016/j.tem.2021.09.004 34654630

[B11] Deng Z. Liu J. Gong H. Cai X. Xiao H. Gao W. (2024). Psychiatric disorders associated with PCSK9 inhibitors: a real-world, pharmacovigilance study. CNS Neurosci. Ther. 30 (4), e14522. 10.1111/cns.14522 37950531 PMC11017405

[B12] Dyer O. (2024). Donanemab: FDA experts recommend approval of Alzheimer's drug. BMJ 385, q1327. 10.1136/bmj.q1327 38876494

[B13] Eimer W. A. Vijaya Kumar D. K. Navalpur Shanmugam N. K. Rodriguez A. S. Mitchell T. Washicosky K. J. (2018). Alzheimer's disease-associated β-amyloid is rapidly seeded by herpesviridae to protect against brain infection. Neuron 99 (1), 56–63. 10.1016/j.neuron.2018.06.030 30001512 PMC6075814

[B14] Espay A. J. Kepp K. P. Herrup K. (2024). Lecanemab and donanemab as therapies for Alzheimer's disease: an illustrated perspective on the data. eNeuro 11 (7), 0319–0323. 10.1523/ENEURO.0319-23.2024 PMC11218032 38951040

[B15] Filippi M. Cecchetti G. Cagnin A. Marra C. Nobili F. Parnetti L. (2023). Redefinition of dementia care in Italy in the era of amyloid-lowering agents for the treatment of Alzheimer's disease: an expert opinion and practical guideline. J. Neurol. 270 (6), 3159–3170. 10.1007/s00415-023-11642-0 36892630 PMC10188416

[B16] Fusaroli M. Raschi E. Poluzzi E. Hauben M. (2024). The evolving role of disproportionality analysis in pharmacovigilance. Expert Opin. Drug Saf. 23 (8), 981–994. 10.1080/14740338.2024.2368817 38913869

[B17] Grabher B. J. (2018). Effects of alzheimer disease on patients and their family. J. Nucl. Med. Technol. 46 (4), 335–340. 10.2967/jnmt.118.218057 30139888

[B18] Heidebrink J. L. Paulson H. L. (2024). Lessons learned from approval of aducanumab for Alzheimer's disease. Annu. Rev. Med. 75, 99–111. 10.1146/annurev-med-051022-043645 38285515 PMC10926277

[B19] Jack C. R. Jr. Bennett D. A. Blennow K. Carrillo M. C. Dunn B. Haeberlein S. B. (2018). NIA-AA Research Framework: toward a biological definition of Alzheimer's disease. Alzheimers Dement. 14 (4), 535–562. 10.1016/j.jalz.2018.02.018 29653606 PMC5958625

[B20] Jiang M. X. Li H. Kong L. T. (2024). Data mining and safety analysis of dual orexin receptor antagonists (DORAs): a real-world pharmacovigilance study based on the FAERS database. Front. Pharmacol. 15, 1436405. 10.3389/fphar.2024.1436405 39166117 PMC11333359

[B21] Kamatham P. T. Shukla R. Khatri D. K. Vora L. K. (2024). Pathogenesis, diagnostics, and therapeutics for Alzheimer's disease: breaking the memory barrier. Ageing Res. Rev. 101, 102481. 10.1016/j.arr.2024.102481 39236855

[B22] Karran E. Strooper B. D. (2022). The amyloid hypothesis in Alzheimer disease: new insights from new therapeutics. Nat. Rev. Drug Discov. 21 (4), 306–318. 10.1038/s41573-022-00391-w 35177833

[B23] Kwon D. (2024). Debate rages over Alzheimer's drug lecanemab as UK limits approval. Nature. 10.1038/d41586-024-02720-y 39179772

[B24] Li D. Wang H. Qin C. Du D. Wang Y. Du Q. (2024). Drug-induced acute pancreatitis: a real-world pharmacovigilance study using the FDA adverse event reporting system database. Clin. Pharmacol. Ther. 115 (3), 535–544. 10.1002/cpt.3139 38069538

[B25] Li J. Wu X. Tan X. Wang S. Qu R. Wu X. (2023). The efficacy and safety of anti-Aβ agents for delaying cognitive decline in Alzheimer's disease: a meta-analysis. Front. Aging Neurosci. 15, 1257973. 10.3389/fnagi.2023.1257973 38020763 PMC10661413

[B26] Li S. Jin M. Liu L. Dang Y. Ostaszewski B. L. Selkoe D. J. (2018). Decoding the synaptic dysfunction of bioactive human AD brain soluble Aβ to inspire novel therapeutic avenues for Alzheimer's disease. Acta Neuropathol. Commun. 6 (1), 121. 10.1186/s40478-018-0626-x 30409172 PMC6225562

[B27] Li Z. Gu J. Du Z. Lu R. Jiang Y. Zhu H. (2025). Characteristics of adverse events and clinical risks of Lecanemab based on FAERS data. J. Affect Disord. 374, 46–54. 10.1016/j.jad.2025.01.022 39793624

[B28] Liu X. Yu C. Yao Y. Lai H. Ye X. Xu J. (2023). Novel neuroprotective pyromeconic acid derivatives with concurrent anti-Aβ deposition, anti-inflammatory, and anti-oxidation properties for treatment of Alzheimer's disease. Eur. J. Med. Chem. 248, 115120. 10.1016/j.ejmech.2023.115120 36682173

[B29] Lopez-Lee C. Torres E. R. S. Carling G. Gan L. (2024). Mechanisms of sex differences in Alzheimer's disease. Neuron 112 (8), 1208–1221. 10.1016/j.neuron.2024.01.024 38402606 PMC11076015

[B30] Ma Y. Liu S. Zhou Q. Li Z. Zhang Z. Yu B. (2024). Approved drugs and natural products at clinical stages for treating Alzheimer's disease. Chin. J. Nat. Med. 22 (8), 699–710. 10.1016/S1875-5364(24)60606-0 39197961

[B31] Mahase E. (2023). Alzheimer's disease: lecanemab gets full FDA approval and black box safety warning. BMJ 382, 1580. 10.1136/bmj.p1580 37419629

[B32] Maheshwari S. Singh A. Ansari V. A. Mahmood T. Wasim R. Akhtar J. (2024). Navigating the dementia landscape: biomarkers and emerging therapies. Ageing Res. Rev. 94, 102193. 10.1016/j.arr.2024.102193 38215913

[B33] Nebel R. A. Aggarwal N. T. Barnes L. L. Gallagher A. Goldstein J. M. Kantarci K. (2018). Understanding the impact of sex and gender in Alzheimer's disease: a call to action. Alzheimers Dement. 14 (9), 1171–1183. 10.1016/j.jalz.2018.04.008 29907423 PMC6400070

[B34] Nguyen H. V. Mital S. Knopman D. S. Alexander G. C. (2024). Cost-effectiveness of lecanemab for individuals with early-stage alzheimer disease. Neurology 102 (7), e209218. 10.1212/WNL.0000000000209218 38484190

[B35] Noguchi Y. Tachi T. Teramachi H. (2021). Detection algorithms and attentive points of safety signal using spontaneous reporting systems as a clinical data source. Brief. Bioinform 22 (6), bbab347. 10.1093/bib/bbab347 34453158

[B36] Prajapati S. K. Pathak A. Samaiya P. K. (2024). Alzheimer's disease: from early pathogenesis to novel therapeutic approaches. Metab. Brain Dis. 39 (6), 1231–1254. 10.1007/s11011-024-01389-6 39046584

[B37] Rabinovici G. D. (2021). Controversy and progress in Alzheimer's disease - FDA approval of aducanumab. N. Engl. J. Med. 385 (9), 771–774. 10.1056/NEJMp2111320 34320284

[B38] Rissman R. A. Langford O. Raman R. Donohue M. C. Abdel-Latif S. Meyer M. R. (2024). Plasma Aβ42/Aβ40 and phospho-tau217 concentration ratios increase the accuracy of amyloid PET classification in preclinical Alzheimer's disease. Alzheimers Dement. 20 (2), 1214–1224. 10.1002/alz.13542 37932961 PMC10916957

[B39] Rong L. Xie M. Jiang M. Qiu H. Kong L. (2024). A post-marketing pharmacovigilance study of avapritinib: adverse event data mining and analysis based on the United States Food and Drug Administration Adverse Event Reporting System database. Br. J. Clin. Pharmacol. 90 (8), 1816–1826. 10.1111/bcp.15673 36702463

[B40] Sato K. Niimi Y. Ihara R. Iwata A. Iwatsubo T. (2024). Adverse events as a cause of unblinding of allocated arms in anti-amyloid therapy trials: a meta-analysis of the predictive value. J. Alzheimers Dis. 101, 1127–1132. 10.3233/JAD-240623 39269842

[B41] Swerdlow N. R. Joshi Y. B. Sprock J. Talledo J. Molina J. L. Delano-Wood L. (2023). Preliminary evidence that memantine enhances prepulse effects on startle magnitude and latency in patients with Alzheimer's disease. J. Alzheimers Dis. 91 (1), 355–362. 10.3233/JAD-220769 36404550 PMC12416332

[B42] Terao I. Kodama W. (2024). Comparative efficacy, tolerability, and acceptability of donanemab, lecanemab, aducanumab, melatonin, and aerobic exercise for a short time on cognitive function in mild cognitive impairment and mild Alzheimer's disease: a systematic review and network meta-analysis. J. Alzheimers Dis. 98 (3), 825–835. 10.3233/JAD-230911 38461503

[B43] Thussu S. Naidu A. Manivannan S. Grossberg G. T. (2024). Profiling aducanumab as a treatment option for Alzheimer's disease: an overview of efficacy, safety and tolerability. Expert Rev. Neurother. 24, 1045–1053. 10.1080/14737175.2024.2402058 39291991

[B44] Varadharajan A. Davis A. D. Ghosh A. Jagtap T. Xavier A. Menon A. J. (2023). Guidelines for pharmacotherapy in Alzheimer's disease - a primer on FDA-approved drugs. J. Neurosci. Rural. Pract. 14 (4), 566–573. 10.25259/JNRP_356_2023 38059250 PMC10696336

[B45] Wu S. Qi Y. Jiang C. Zheng J. (2025). Mining and analysis of adverse events associated with aducanumab: a real-world study using FDA Adverse Event Reporting System database. Expert Opin. Drug Saf. 24, 469–478. 10.1080/14740338.2024.2448205 39726994

[B46] Wu W. Ji Y. Wang Z. Wu X. Li J. Gu F. (2023). The FDA-approved anti-amyloid-beta monoclonal antibodies for the treatment of Alzheimer's disease: a systematic review and meta-analysis of randomized controlled trials. Eur. J. Med. Res. 28 (1), 544. 10.1186/s40001-023-01512-w 38017568 PMC10683264

[B47] Xing X. Zhang X. Wang K. Wang Z. Feng Y. Li X. (2025). Post-marketing safety concerns with lecanemab: a pharmacovigilance study based on the FDA Adverse Event Reporting System database. Alzheimers Res. Ther. 17 (1), 15. 10.1186/s13195-024-01669-4 39780222 PMC11708074

[B48] Xiong B. Song Z. Wang L. Zhang A. Zhou Y. Zheng N. (2024). Can targeted protein degradation technology provide a potential breakthrough in the development of anti-AD drugs? ACS Chem. Neurosci. 15 (19), 3434–3436. 10.1021/acschemneuro.4c00590 39354828

[B50] Yang Y. Qiu L. (2024). Research progress on the pathogenesis, diagnosis, and drug therapy of Alzheimer's disease. Brain Sci. 14 (6), 590. 10.3390/brainsci14060590 38928590 PMC11201671

[B51] Zhang J. Zhang Y. Wang J. Xia Y. Chen L. (2024). Recent advances in Alzheimer's disease: mechanisms, clinical trials and new drug development strategies. Signal Transduct. Target Ther. 9 (1), 211. 10.1038/s41392-024-01911-3 39174535 PMC11344989

[B52] Zuliani G. Zuin M. Romagnoli T. Polastri M. Cervellati C. Brombo G. (2024). Acetyl-cholinesterase-inhibitors reconsidered. A narrative review of post-marketing studies on Alzheimer's disease. Aging Clin. Exp. Res. 36 (1), 23. 10.1007/s40520-023-02675-6 38321321 PMC10847178

